# Superficial Femoral Artery Recanalization Using Fiber Optic RealShape Technology

**DOI:** 10.3390/medicina58070961

**Published:** 2022-07-20

**Authors:** Jurre Klaassen, Joost A. van Herwaarden, Martin Teraa, Constantijn E. V. B. Hazenberg

**Affiliations:** Department of Vascular Surgery, University Medical Center Utrecht, 3584 CX Utrecht, The Netherlands; j.klaassen-4@umcutrecht.nl (J.K.); j.a.vanherwaarden@umcutrecht.nl (J.A.v.H.); m.teraa@umcutrecht.nl (M.T.)

**Keywords:** endovascular surgery, superficial femoral artery, occlusion, recanalization, fiber optic technology, three dimensional, radiation

## Abstract

Purpose: Report of a successful case of endovascular recanalization of an occluded superficial femoral artery (SFA) using Fiber Optic RealShape (FORS) technology. Case Report: A 79-year-old male was referred for evaluation of multiple ischemic pretibial ulcers of the right lower extremity. Computed tomography–angiography (CTA) imaging confirmed significant stenosis of the right common femoral artery (CFA) and an occlusion of the SFA from its origin to the Hunter’s canal. The patient was treated with a hybrid surgical procedure: an endarterectomy of the CFA and SFA origin was performed combined with an endovascular recanalization of the occluded SFA using FORS technology. During recanalization, the FORS guidewire slowly twisted subintimally around the occluded lumen of the SFA, maintaining the created corkscrew shape after pre-dilation with the percutaneous transluminal angioplasty (PTA) balloon and subsequent stenting. Conclusions: FORS technology can be successfully used during recanalization of an occluded SFA without the use of fluoroscopy. The corkscrew shape formed during recanalization in this case was retained during PTA balloon pre-dilation and stenting; this potentially improves hemodynamics and thereby reduces the risk of in-stent restenosis. However, expanding patient series and longer follow-up data are needed to increase the understanding of the feasibility and effectiveness of using FORS in the treatment of peripheral arterial occlusive disease.

## 1. Introduction

Exposure to fluoroscopy during endovascular procedures poses risks to both the patient and the treatment team [1,2,3]. In addition, three-dimensional structures are projected onto screens only as two-dimensional grayscale images, eliminating spatial perception of depth and tortuosity. To reduce the use of fluoroscopy and improve spatial perception, while navigating during endovascular procedures, Philips (Philips Koninklijke N.V., Best, The Netherlands) has developed Fiber Optic RealShape (FORS) technology in collaboration with clinical experts. FORS combines image fusion with specially designed endovascular devices with embedded optical fibers that use laser light instead of fluoroscopy for three-dimensional visualization of guidewires and catheters. The FORS technology, outcomes of the preclinical study and outcomes of the first in human feasibility study, have been published and described previously [4,5]. In this case report, we describe a successful recanalization of an occluded superficial femoral artery (SFA) using FORS technology, which provided greater insight into the spatial course of the devices during recanalization without the need for fluoroscopy.

## 2. Case Report

A 79-year-old male was referred for evaluation of multiple ischemic pretibial ulcers (W1I3fI1) of the right lower extremity (Rutherford stage 5 classification). The patient had a history of smoking, cerebrovascular disease, hypertension, renal insufficiency, and endovascular recanalization of the right common iliac (CIA) and left external iliac artery (EIA) combined with bilateral thromboendarterectomy (TEA) of the common femoral artery (CFA). The photoelectric plethysmography toe-arm index was 0.34 and duplex ultrasound showed patency of previously placed stents in the CIA and EIA, however a flow-limiting stenosis in the right CFA and occlusion of the SFA were observed. Computed tomography–angiography (CTA) imaging confirmed significant stenosis of the right CFA and occlusion of the SFA from its origin to the Hunter’s canal.

**Figure 1:**
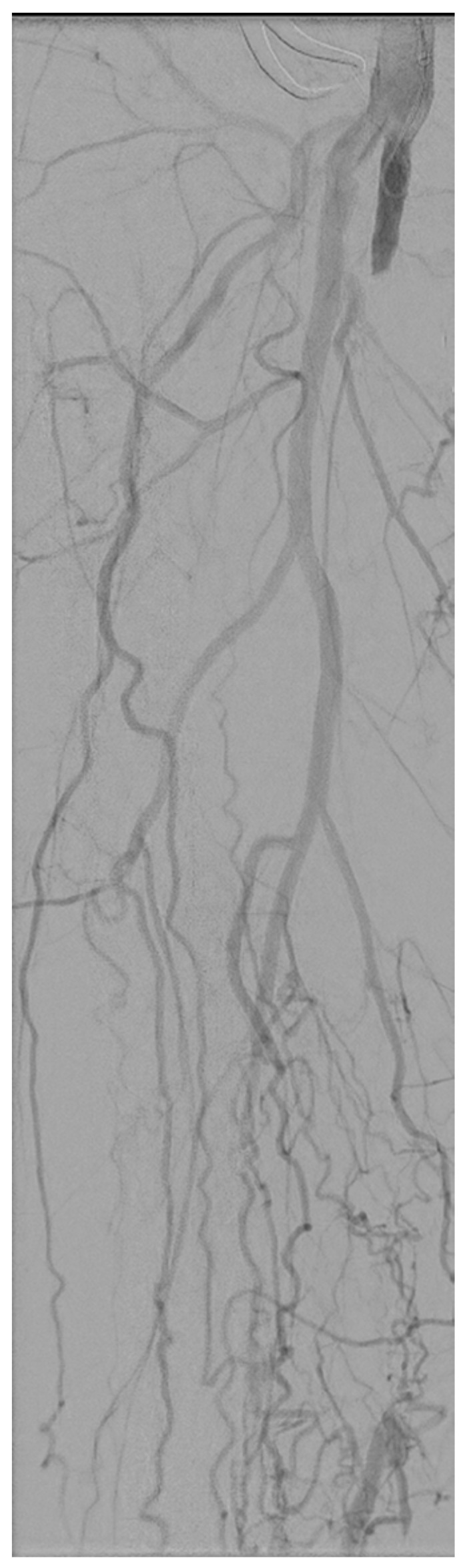
Overview of the baseline angiography of the left superficial femoral artery (SFA) showing that the SFA is largely occluded.

**Figure 2:**
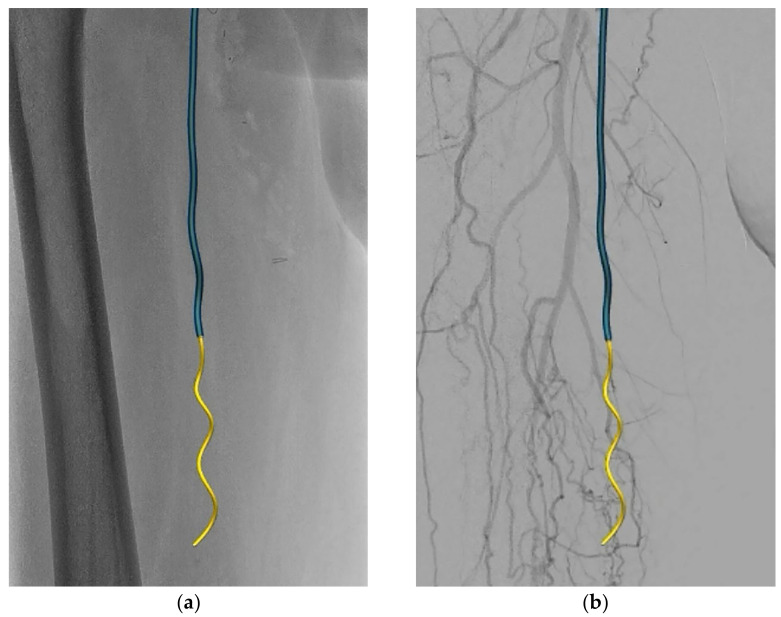
Overview of recanalization of the occluded SFA with fluoroscopic (**a**) and digital substraction angiography (DSA) (**b**) background using 0.035 inch angled hydrophilic floppy Fiber Optic Realshape (FORS) enabled guidewire (yellow) and 5.5F FORS-Berenstein catheter (blue). The three-dimensional (3D) visualization of the FORS enabled guidewire clearly shows that the guidewire, with the tip pointing forward, slowly twisted subintimally around the occluded SFA like a corkscrew.

**Figure 3:**
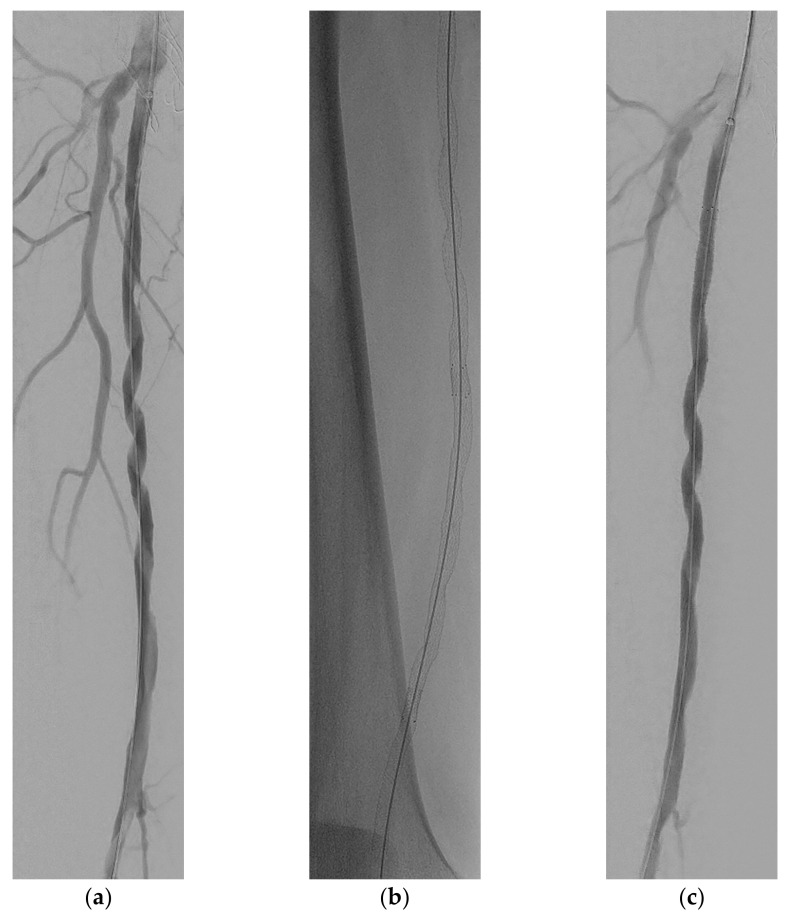
Overview of post percutaneous transluminal angioplasty (PTA) balloon dilatation DSA (**a**) and self-expandable (SE) stent placements result (**b**) and completion DSA (**c**). All three images show a similar corkscrew shape as the FORS enabled guidewire showed during recanalization of occluded SFA (Figure 2).

The treatment team decided to treat the abovementioned vascular lesions in a hybrid surgical procedure: an endarterectomy of the CFA and SFA origin combined with an endovascular recanalization of the occluded SFA using FORS technology. The patient provided informed consent for the procedure. The CFA was approached via a standard surgical approach and the atherosclerotic plaque was removed via open endarterectomy with venous patch closure. Subsequently, the venous patch was punctured and a 6F sheath was placed over a standard guidewire. Digital subtraction angiography (DSA) was performed and confirmed the SFA occlusion (Figure 1). Furthermore, the FORS-enabled 0.035 inch angled hydrophilic floppy guidewire and 5.5F FORS-Berenstein catheter were registered out of body for use. During recanalization of the occluded SFA, the three-dimensional (3D) visualization of the FORS-guidewire clearly showed that the guidewire was following the path of least resistance as the guidewire, with the tip pointing forward, slowly twisted subintimally around the occluded SFA like a corkscrew (Figure 2). At the popliteal segment P1, re-entry to the true lumen was obtained. The entire tract was predilated with a 5 mm × 200 mm plain percutaneous transluminal angioplasty (PTA) balloon (Abbott Laboratories, Lake bluff, Illinois, United States) and then treated with two 6 mm × 150 mm drug-coated balloons (DCB) (Cardionovum GmbH, Bonn, Germany). A post dilatation DSA was created to assess the recanalized SFA and to assess any residual flow-limiting dissections or stenosis, which showed a remaining dissection in the P1 segment (Figure 3a). First, this dissection was treated with a 5 mm × 60 mm PTA balloon. However, post PTA DSA still showed a significant dissection in the P1 segment, which was treated with a 5 mm × 60 mm Supera stent (Abbott Laboratories, Lake bluff, Illinois, United States) and extended to the proximal SFA with two 6 mm × 150 mm self-expandable (SE) Everflex stents (Medtronic, Dublin, Ireland) because of remaining dissection (Figure 3b). Finally, control DSA was performed in two different planes, which showed no residual stenosis or dissection, but a persistent corkscrew configuration (Figure 3c). Skin-to-skin procedure time was 367 min, of which 194 min was endovascular recanalization time. In addition, during the procedure the air kerma (AK) was 57.14 mGy, the dose area product (DAP) was 14.93 Gy/cm^2^, and the fluoroscopy time (FT) was 1046 s.

After the procedure acetylsalicylic acid 80 mg daily was added to clopidogrel 75 mg once daily and continued for three months after which only clopidogrel was continued. After the procedure, wounds healed within 6 weeks and duplex ultrasound after 6 months confirmed patency of the treated segment without restenosis.

## 3. Discussion

This report presents the first ever described case of successful recanalization of an occluded SFA using FORS technology. Using 3D visualized FORS-enabled devices, it was apparent during recanalization that the guidewire slowly twisted subintimally around the occluded lumen of the SFA. In addition, during pre-dilation with the PTA balloon and placement of the SE stents, these devices maintained the corkscrew shape formed during recanalization.

**Figure 4:**
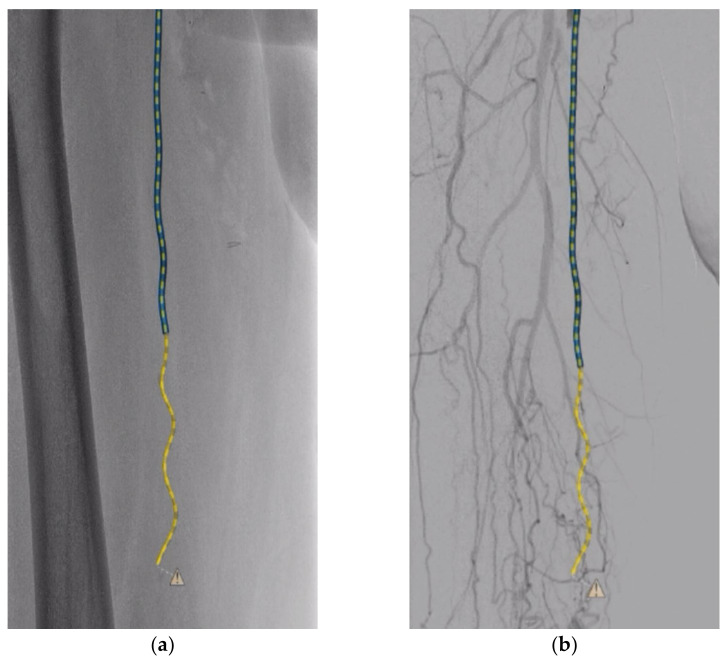
Example of FORS guidewire visualization with dashed line during recanalization of the occluded SFA with fluoroscopic (**a**) and DSA (**b**) background. The FORS technology visualizes devices with a dashed line during a period when excessive force on the guidewire impairs the optical signal, exceeding the boundary conditions for guidewire shape reconstruction.

FORS technology has been developed to reduce the use of fluoroscopy and improve spatial perception while navigating during endovascular procedures. As a result, FORS appears to be of particular value during navigation in, for example, complex endovascular repairs of aortic aneurysms or complex lesions in tortuous iliac arteries, and perhaps less so in this near-straight SFA occlusion. However, the use of FORS technology is also of added value in this case because the recanalization, with the exception of creating the re-entry, was performed without the use of fluoroscopy. Radiation exposure and related parameters in femoropopliteal interventions are significantly influenced by lesion length and complexity, which complicates direct comparisons of this single case to literature examples. Literature that reported on radiation use in femoropopliteal interventions mainly included TASC A or B lesions, which are less complex than the TASC D lesion described in this case, and did not report separate data on complex lesions [6,7,8,9,10,11]. Moreover, FORS technology is currently limited to improving endovascular navigation and it would be better to look specifically at the radiation-reducing effect at this stage of the procedure. The recanalization (start to reentry) was performed in 74 min and the radiation exposure parameters for this phase of the procedure comprised an AK of 12 mGy, a DAP of 3.71 Gy/cm^2^, and an FT of 557 s. Predilation treatment with DCBs, stent placement, and control DSAs accounted for the remainder of the procedural radiation exposure parameters. While FORS was used in the majority of the time to create the recanalization tract (56 min), the amount of FT was relatively lower during FORS usage (103 s) than when FORS was not used (454 s). Additionally, during FORS usage, the AK and DAP were 2 mGy and 0.82 Gy/cm^2^ and can be attributed to the use of DSAs used to create roadmaps and ensure the accuracy of the FORS devices during recanalization. The use of FORS technology therefore enables the practitioner to perform these types of procedures while reducing radiation exposure and thus the potential health risks associated with it, such as DNA damage and long-term health effects. Moreover, FORS technology provides feedback on the force applied to the guidewire, in addition to standard tactile feedback. Excessive force on the guidewire will impair its optical signal, exceeding the boundary conditions for reconstruction of the shape of the guidewire. Therefore, the FORS technology visualizes the entire guidewire as a dashed line rather than a solid line during the period when excessive force is applied to it (Figure 4). In this way the practitioner receives feedback in two ways when too much force is applied on the devices during the recanalization. In a training setting, it also offers the supervising practitioner the opportunity to gain more insight into the force exerted during recanalization and to intervene, if necessary, in high-risk situations.

Subintimal angioplasty (SA) for recanalization of chronic total occlusions of the femoro-popliteal tract was first described by Bolia et al. [12] and aims to cross occlusive lesions subintimally. During SA, the guidewire is used in a loop configuration to advance along the natural dissection plane of the occluded SFA [13]. However, compared to a commonly used conventional hydrophilic nitinol Terumo Radiofocus M guidewire (Terumo, Shibuya, Tokio, Japan), the first release FORS-enabled guidewire has slightly stiffer properties along the entire length, which impact the formation of a loop. As a result, during recanalization with FORS technology, the guidewire was able to be advanced subintimally with the tip facing forward, probably following the path of least resistance, resulting in a corkscrew-like shape, as described in this case and shown in Figure 2. In this first case the FORS wire passed the occlusion very smoothly without signs of perforation.

During predilatation of the recanalized SFA pathway with a PTA balloon and subsequent placement of the SE stents, these devices showed a similar corkscrew shape as the FORS guidewire during recanalization. Although the fact that the corkscrew shape is not necessary for the consequence of the FORS characteristics, this corkscrew configuration may provide hemodynamic benefits as spiral laminar flow appears to be the preferential arterial flow pattern [14]. Since recent studies have shown that changes in wall shear stress caused by local hemodynamic stent-induced alterations are a potential cause of in-stent restenosis, placement of stents that maintain spiral laminar flow may provide a solution compared to conventional stents [15,16,17]. For example, 2-year results from the MIMIC-2 trial show that the swirling flow BioMimics 3D vascular stent is safe and showed no major limb amputations with sustained clinical improvement in walking distance, walking speed and stair climbing [18]. Although we essentially used straight SE stents in this case, the shape of the recanalized trajectory gives them a spiral shape comparable to the BioMimics stent, potentially allowing them to have similar hemodynamic properties and benefits. However, additional research on fluid dynamics, longer follow-up, and more similar case studies are needed to verify this hypothesis.

While the use of FORS-enabled guidewires during recanalization provides improved 3D perception of the trajectory and recanalization could be performed with laser-light navigation instead of using fluoroscopy, there are also limitations to their use. For example, the current FORS software (Release 1) is unable to visualize a narrow looped (<6 mm radius) FORS guidewire without the use of fluoroscopy. As a result, FORS visualization of the FORS guidewire is only possible if the guidewire is advanced with the tip facing forward which may increase the risk of perforation during recanalization. In addition, only the 0.035 inch angled hydrophilic FORS-enabled guidewire with a working length of 120 cm is currently available. As a result, the FORS technology is currently not applicable when performing recanalizations below the knee. Further development of the current guidewire and increasing the number of available guidewires and catheters is therefore necessary to broaden the application of FORS.

## 4. Conclusions

This report is the first to describe a case of a successful recanalization of an occluded SFA using FORS technology. As a result, with the exception of creating a re-entry, the recanalization was performed without using fluoroscopy. The deployed SE stents adopted a corkscrew shape similar to that of the guidewire during recanalization, potentially improving hemodynamics and reducing the risk of in-stent restenosis. However, expanded patient series and longer follow-up data are needed to increase understanding of the feasibility and radiation dose reduction effectiveness of using FORS in the treatment of peripheral arterial occlusive disease.

## Data Availability

The data presented in this study is available on request from the corresponding author. The data is not publicly available due to privacy restrictions.
